# Effect of Dietary Organic and Inorganic Sulfur on the Performance of Coccidiosis Vaccine Challenged Broiler Chickens

**DOI:** 10.3390/ani12091200

**Published:** 2022-05-06

**Authors:** Yong-Sung Jeon, Yoo-Bhin Kim, Hyun-Gwan Lee, Jina Park, Yun-Ji Heo, Gyo-Moon Chu, Kyung-Woo Lee

**Affiliations:** 1 Department of Animal Science and Technology, Konkuk University, 120 Neungdong-ro, Gwangjin-gu, Seoul 05029, Korea; fmdyd94@kakao.com (Y.-S.J.); ybin51@naver.com (Y.-B.K.); leehyun3177@naver.com (H.-G.L.); jinaa97@konkuk.ac.kr (J.P.); bye0604@naver.com (Y.-J.H.); 2 Busanbio, Nonghyup Feed Co., Ltd., 337 Uam-ro, Nam-gu, Busan 48475, Korea; gyomoon96@hanmail.net

**Keywords:** sulfur, antioxidants, coccidiosis, growth performance, broiler chickens

## Abstract

**Simple Summary:**

Coccidiosis is a parasitic disease in poultry that causes significant economic losses. It is understood that natural or synthetic antioxidants including plant extracts, vitamin E, and selenium have been proved to lessen the gut severity of *Eimeria* infection in chickens. Sulfur is an essential element and exhibits beneficial activities including parasitic, antioxidant, and anti-inflammatory. These functional features of sulfur might play a role in inhibiting the negative effect of chicken coccidiosis and, if proved, sulfur could be used as an anticoccidial agent. We tested dietary sulfur of both organic and inorganic forms with beneficial antioxidant properties in a mild coccidiosis disease broiler chicken model.

**Abstract:**

The objective of this study was to evaluate the effects of dietary sulfur from either organic (methyl sulfonyl methane, MSM) or inorganic (sodium sulfate, SS) sources on the growth performance of broiler chickens challenged against a high-dose coccidiosis vaccine. A total of 320 day-old Ross 308 broiler chicks were randomly placed into 32 pens of 10 birds each (keeping 16 pens/control group and 8 pens/treatment group until 21 days post-hatch) and reared for 28 days. The experimental diets were formulated by mixing a corn and soybean meal-based control diet with MSM or SS. At 21 days post-hatch, half (*n* = 8) of the control and all of the sulfur-added diet-fed (i.e., MSM and SS) groups were challenged with a 30-fold dose of a commercially available *Eimeria* vaccine (Livacox^®^ T coccidiosis vaccine). Unchallenged control chicks (*n* = 8) were considered as the negative control group. At 21 days (before coccidiosis vaccine challenge), the production parameters and cecal short-chain fatty acids were not affected by dietary treatments. The concentrations of total antioxidant capacity in liver samples were elevated in both the MSM and SS groups compared with the control group (*p* = 0.001). During 21 to 28 days (i.e., one week post coccidiosis vaccine challenge), challenge tended to lower body weight and feed intake by an average of 5.3% (*p* = 0.262) and 2.8% (*p* = 0.504), respectively, but to increase the feed conversion ratio by an average of 2.7% (*p* = 0.087) compared with the non-challenged control groups. None of dietary sulfur groups affected the body weight gain, feed intake, or feed conversion ratio of vaccine-challenged chickens. Mild *Eimeria*-specific lesions were noted in duodenum (*p* = 0.006), jejunum (*p* = 0.017), and ceca (*p* = 0.047), but dietary sulfur treatments did not affect the *Eimeria*-induced gut lesion scores. At 28 days, *Eimeria* challenge significantly impaired (*p* = 0.001) the apparent ileal digestibility of crude protein and crude ash compared with the naïve control group. Dietary MSM increased the apparent ileal digestibility of crude ash by 15.5% on average compared with the coccidiosis vaccine control group. We conclude that dietary antioxidant sulfur of organic or inorganic origins at the inclusion level (i.e., 0.7 g sulfur/kg of diet) has a limited effect on the growth performance of chickens challenged with coccidiosis vaccine.

## 1. Introduction

Coccidiosis is an enteric disease affecting the performance, welfare, and health of chickens with an estimated economic loss of approximately GBP 10.36 billion in the global poultry industry [1]. It is caused by several species of the genus *Eimeria* that are known to invade specific sites of the gastrointestinal tract, inducing mild to severe gut lesions [2]. Although anticoccidial medications have been used to control coccidiosis, concerns about the occurrence of resistant *Eimeria* spp. and the residue on poultry meats have led the efforts to develop alternative nutritional strategies including probiotics, prebiotics, phytochemicals, and antimicrobial peptides [3]. These potential alternative candidates may share working mechanisms such as reduction in the pathogenic (i.e., oocysts) load, mitigation of oxidative stress-induced gut damage, or enhancement of intestinal protective immunity in chickens [2]. Recently, in-feed natural or synthetic antioxidants have been shown to lessen the severity of chicken coccidiosis, as *Eimeria* infection is associated with lipid peroxidation in the intestinal mucosa [4]. Among the antioxidants tested, plant extracts [5], vitamin E [6], and selenium [7] are known to control avian coccidiosis.

Sulfur is an essential element for the growth of most animals including humans [8]. Sulfur per se is not stored in the body, but animal diets need a supply of sulfur-containing macromolecules [9]. In addition, sulfur has been used as an antimicrobial/anticoccidial agent for treating bacterial diseases and chicken coccidiosis due to its parasite killing, antioxidant, and immune-modulating activity [10,11,12]. Chickens are generally resistant to the sulfur toxicity; sulfur tolerance is reported to be 14,000 ppm for broilers and 8100 ppm for laying hens [13]. It is thus understood that sulfur toxicity in chickens is hardly seen at the commercial setting, and sulfur can be provided via consuming sulfur-containing water and ingredients or various forms of sulfate minerals.

Methyl sulfonyl methane (MSM) is an organic sulfur naturally found in all living organisms including insects, plants, animals, and humans [14,15]. It is an oxidized metabolite of dimethyl sulfoxide and contains 34% sulfur on a weight basis [16]. MSM has been known to exhibit anti-inflammatory and antioxidant activities in vitro [14] and in vivo [17]. In addition, it is effective in treating parasitic infection by *Trichomonas vaginalis* or *Giardia lamblia* in animals including humans [18]. The biological and parasitic activities of MSM make it a promising anticoccidial agent in chickens by inhibiting parasitic growth, augmenting preventive immunity, or mitigating parasite-induced oxidative stress. However, Abdul Rasheed et al. [19] failed to see the anticoccidial effect of MSM in *Eimeria* infected chickens. They noted an increase in oocyst counts and feed conversion ratios but a decrease in oxidative stress in the plasma samples of MSM-fed chickens compared with the *Eimeria*-infected control group. Interestingly, MSM stimulated feed intake without affecting body weight gain compared with the infected control group. Thus, the lacking effect of dietary MSM or sulfur-containing molecules on avian coccidiosis remains to be concluded. When considering the antiparasitic effect of sulfur [12,18], we decided to re-test dietary MSM as a potential anticoccidial agent in broiler chickens. In addition to MSM as an organic sulfur, we included sodium sulfate (SS) as an inorganic sulfur to compare the effectiveness of different forms of sulfur in broiler chickens. It has been reported that both MSM and SS exhibit antimicrobial and antioxidant properties in laying hens [20]. Nonetheless, Kim et al. [20] concluded that SS vs. MSM might have different actions on feed or water intake, indicating different biological fates or functions between organic and inorganic sulfur sources in laying hens. Taken together, it would be of value to evaluate different forms of sulfur in a mild coccidiosis broiler chicken model.

## 2. Materials and Methods

### 2.1. Birds and Experimental Design

**Figure 1:**
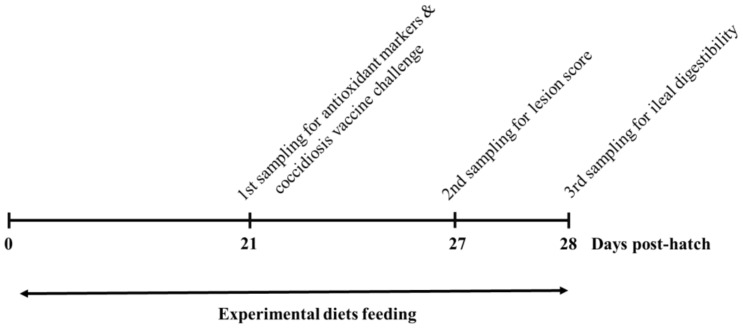
Schematic outline of the experimental design. Chickens were fed diets containing none, methyl sulfonyl methane, or sodium sulfate until the end of the experiment. Blood, liver, and intestine samples were obtained at 21 days, and all chickens except for the non-challenged control groups were orally challenged with coccidiosis vaccine at 21 days. Intestinal samples were obtained for scoring gut lesions at 27 days (i.e., 6 days of post vaccine challenge). At 28 days, ileal digesta were sampled for assessing ileal nutrient digestibility.

A total of 320 day-old broiler chicks (Ross 308) were obtained from a local hatchery. Upon arrival, they were individually weighed, randomly placed into 32 floor pens with fresh rice husks as a bedding material, and subjected to one of three dietary treatments. Each pen measured 1 m in width and 2 m in length and had 10 birds per pen. The control group kept 16 pens until 21 days, while the experimental groups had 8 pens. The experimental timeline outlined in Figure 1.

A corn-soybean meal-based diet was prepared and used as a control diet (Table 1). The experimental diets were formulated by mixing the control diet with MSM (Sigma-Aldrich, St. Louis, MO, USA) or SS (Samchun Chemicals, Pyeongtaek, Korea) at concentrations of 2.0 g MSM/kg and 3.0 g of SS/kg to provide 0.7 g sulfur, either organic or inorganic, per kg of diet. As SS contains sodium, sodium bicarbonate was used to meet equal sodium contents in all experimental diets. The MSM contents in the basal and experimental diets were measured using gas chromatography as described by Park and Lee [21].

At 21 days, half of the control groups (*n* = 8/treatment, positive control) and all of the MSM/SS groups were orally gavaged with 30× the recommended dose of the attenuated Livacox^®^ T coccidiosis vaccine (Biopharm, Jilove u Prahy, Czech Republic) to induce mild coccidiosis [19,22]. Broilers not vaccinated with the coccidiosis vaccine were considered as unchallenged negative control chickens. The feed intake and body weight by per pen were monitored weekly to calculate the feed conversion ratio. The broiler facility was initially set at 32 °C, then gradually decreased to reach 24 °C at 21 days, and kept constant thereafter. The light was set with one-hour darkness per day.

### 2.2. Sampling

At 21 days, 1 bird per pen (8 chicks per treatment) was euthanized by carbon dioxide. Immediately after euthanasia, blood was sampled via heart puncture. Serum samples were obtained by gentle centrifugation at 200× *g* for 15 min [20] and stored at −20 °C until use. Then, ileum, ceca, and liver were sampled. Cecal digesta were collected to measure the concentrations of short-chain fatty acids. Ileal mucosa samples were obtained by scraping the mucosa of a 5-cm long mid-ileum segment. The obtained mucosa scrapings were washed with ice-cold 1 × PBS and centrifuged at 4 °C at 1200× *g* for 10 min, and the supernatants were stored at below 20 °C until use. At 27 days (6 days post coccidiosis vaccine challenge), 1 bird per pen (8 chicks per treatment) was euthanized and intestinal samples (i.e., duodenum, jejunum, ceca) were sampled to score *Eimeria* specific lesions.

### 2.3. Measurement of Volatile Fatty Acids

Approximately 1 g cecal content sampled at 21 days was suspended in 9 mL cold distilled water, and the suspension was mixed with 0.05 mL saturated HgCl_2_, 1 mL 25% H_3_PO_4_, and 0.2 mL 2% pivalic acid. The mixture was centrifuged at 1000× *g* at 4 °C for 20 min and the supernatant collected to measure the concentrations of short-chain fatty acid (SCFA) using gas chromatography (6890 Series GC System, HP, Palo Alto, CA, USA) as described by Kim et al. [23].

### 2.4. Lesion Scores

Approximately 20-cm-long mid-segments of duodenum, jejunum, and whole cecum sampled at 27 days (i.e., 6 days post vaccine infection) were taken and cut longitudinally. Intestinal contents were gently removed and lesion scores from 0 to 4 in range of severity as described elsewhere [24], were independently made by 3 observers in a blinded fashion.

### 2.5. Measurement of Antioxidant Parameters

Serum, liver, and ileum mucosa samples were used to analyze the antioxidant parameters. The diluted samples were used for the determination of total antioxidant capacity (TAC) (QuantiChrom^TM^ antioxidant assay kit-DTAC 100, BioAssay Systems, Hayward, CA, USA) and malondialdehyde (MDA)(OxiSelect TBARS Assay kit, Cell Biolabs, Inc., San Diego, CA, USA). The protein concentrations in liver and ileal mucosa samples were determined by the Bradford assay procedure using bovine serum albumin as the standard [25]. The color was measured at 595 nm using an ELISA plate reader (Bio-Rad, Model 550, Hercules, CA, USA).

### 2.6. Nutrient Digestibility

From days 21 to 28 post-hatch, broilers were fed the diets mixed with 2% celite as an indigestible marker to determine the apparent ileal digestibility (AID) of nutrients. At 28 days (i.e., 7 days post coccidiosis vaccine challenge), all birds per pen were killed by carbon dioxide. Immediately after euthanasia, ileal digesta were sampled by gentle finger stripping of the ileal segment and pooled per pen. The pooled samples were used for chemical analysis of nutrients. Feed and digesta samples were analyzed for insoluble ash [26], dry matter (DM) [27], crude protein (CP) [27], and crude ash [27]. The apparent ileal digestibility of the nutrients was calculated using the following equation:

AID = 100 − [(feed indicator %/ileal indicator %) × (ileal nutrient %/feed nutrient %) × 100]

### 2.7. Statistical Analysis

Each pen was considered as an experimental unit. Data were initially checked for normality using PROC UNIVARIATE (version 9.4; SAS Institute Inc., Cary, NC, USA) and analyzed by one-way ANOVA using the PROC GLM (version 9.4; SAS Institute Inc., Cary, NC, USA). The Tukey test was used to determine the differences among treatments as a post hoc test after ANOVA. The significance level was preset at *p* < 0.05.

## 3. Results

### 3.1. MSM and Sulfur Contents in the Control and Experimental Diets

MSM and SS were added into the control diet to reach concentrations of 2.0 g MSM and 3.0 g SS per kg of diet, which corresponds to 0.7 g sulfur per kg of each diet (Table 2). The sulfur contents in the control, MSM, and SS diets were analyzed to contain 3.52 g, 4.20 g, and 4.21 g sulfur per kg of diet. In addition, the MSM contents were analyzed to contain 1.71 g MSM/kg of diet. Interestingly, the control and SS diets had a low but detectable 0.28–0.29 g MSM per kg of diet, indicating the natural presence of MSM in the feed ingredients used.

### 3.2. Growth Performance before and after Eimeria Vaccine Challenge

At 21 days, none of the dietary treatments affected (*p* > 0.05) body weight gain, feed intake, or feed conversion ratio in naïve broiler chickens (Table 3). Importantly, dietary MSM tended to lower the body weight gain and feed intake by 3.2% and 2.6% on average compared with the control group at 21 days. At day 21, all broiler chickens except for half of the control group were challenged against the coccidiosis vaccine to see, if any, the anticoccidial effect of dietary sulfur in broiler chickens. *Eimeria* vaccine challenge numerically tended to adversely affect body weight, feed intake, and feed conversion ratio by an average of 5.3% (*p* = 0.262), 2.8% (*p* = 0.504), and 2.7% (*p* = 0.087) compared with the non-challenged control groups. However, dietary sulfur failed to affect the growth performance of broiler chickens challenged with coccidiosis vaccine (Table 4), although dietary MSM tended to lower the body weight gain and feed intake by an average of 2.6% and 2.0% compared with the challenged control group.

### 3.3. Concentration of SCFAs in Cecal Digesta

At 21 days (before *Eimeria* vaccine challenge), SCFAs in cecal digesta were monitored to investigate the effect of dietary sulfur on distal fermentation profiles in naïve chickens. Acetate is the most dominant SCFA, followed by butyrate, and propionate in all treatments (Table 5). However, none of the dietary treatments affected the absolute and relative concentrations of SCFAs in cecal digesta, although the highest relative BCFA concentration was shown in the MSM group.

### 3.4. Antioxidant Markers in Serum, Liver, and Ileal Mucosa Samples

At 21 days (before *Eimeria* vaccine challenge), the TAC concentrations in liver samples were increased in both the MSM and SS groups compared with the control group (*p* = 0.001) (Table 6). However, the MSM and SS groups did not affect the TAC concentrations in serum and ileal mucosa samples. As a biomarker of oxidative stress, MDA levels were monitored in serum, liver, and ileal mucosa scrapings. However, none of the dietary treatments affected the MDA levels in the assayed samples.

### 3.5. Gut Lesion Score

Gut lesions at the duodenum, jejunum, and ceca were scored at 6 days post *Eimeria* vaccine challenge. No *Eimeria*-specific lesions were noted in the unchallenged control group (Table 7). As expected, *Eimeria*-specific, but mild, lesions were noted in the duodenum, jejunum, and ceca of the challenged groups. Chickens fed the diets containing MSM and SS exhibited the highest, but non-significant, duodenal lesions compared with the challenged control group. Jejunal lesions were the highest in the challenged control group and were intermediate in both the MSM and SS groups. MSM-fed chickens had the highest, but non-significant, cecal lesions compared with the challenged control group. It was however apparent that all challenged chickens produced mild lesions.

### 3.6. Ileal Nutrient Digestibility

At 28 days (i.e., 7 days post *Eimeria* vaccine challenge), the effect of dietary sulfur on the AID of nutrients was monitored. *Eimeria* vaccine challenge significantly affected the AID of CP and crude ash compared with the naïve control group (Table 8). The MSM and SS did not affect the *Eimeria*-induced decrease in the AID of crude protein. However, the MSM, but not the SS, group increased the AID of crude ash by an average of 15.5% compared with the challenged control group. Neither *Eimeria* challenge nor dietary sulfur affected the AID of dry matter.

## 4. Discussion

It is clear from this study that dietary organic or inorganic sulfur did not affect the growth performance of broiler chickens or those challenged with a coccidiosis vaccine. The lack of effect of MSM and SS on the naïve or coccidiosis vaccine-challenged broilers may not be due to the low addition level, the absence of a biological effect (e.g., antioxidant), or a failure in reproducing the coccidiosis disease model. On the contrary, dietary MSM exhibited a negligible tendency for a decrease in the performance of broilers in both the pre- and post-infection periods, indicating a low statistical power. In this study, we confirmed the antioxidative effect of MSM and SS as manifested by elevating the TAC concentration in liver samples of broiler chickens, which agrees with earlier studies in broiler chickens [17,19] and laying hens [20]. The added levels of MSM and SS (i.e., 0.07% sulfur in diets) were effective in exhibiting the antioxidative effect in laying hens [20]. In line with our findings, no effect of dietary MSM on productive performance was found in naïve ducks [28], naïve broiler chickens [17], or *Eimeria* vaccine-challenged broilers [19]. In contrast to our findings, increasing dietary MSM from 0.05 to 0.3% improved body weight gain and feed conversion ratio, but not feed intake, in broiler chickens [29] and Pekin duck [30]. It was found that those studies with MSM-induced improvement in growth performance were associated with an increase in immunity and antioxidant parameters coupled with a shift in gut microbiota [29]. In this study, we used the coccidiosis vaccine challenge model to evaluate the protective effect of MSM and SS, if any, in broiler chickens. It is well reported that a high dose of coccidiosis vaccine challenge has been known to induce experimental coccidiosis with varying degree of gut lesions in broiler chickens [19,23]. We reproduced mild coccidiosis with an attenuated coccidiosis vaccine as manifested by *Eimeria*-specific mild lesions on duodenum, jejunum, and cecum and the moderate reduction in growth performance. Thus, the anticoccidial efficacy of MSM or SS on chicken coccidiosis would have been detected, if present, in this challenge model. Abdul Rasheed et al. [19] reported that dietary MSM did not affect performance, gut lesions, and fecal oocyst output in a mild coccidiosis disease chicken model. They speculated that mild *Eimeria* infection might not be sufficient to disclose the anticoccidial effect, if any, of dietary MSM in broiler chickens. At this stage, a clear explanation on the lack of effect of MSM or SS on chicken coccidiosis is not readily available; that needs to be addressed. However, as the chicken exhibits resistance to sulfur toxicity [13], higher inclusion doses in a clinical coccidiosis model using different doses or *Eimeria* field strains might be needed in future studies. In addition, the clinical vs. sub-clinical disease model would be considered the better experimental model for testing potential anticoccidial agents as the former vs. the latter could increase the treatment effect size, and such practice could reduce the number of animals without lowering the statistical power.

The recent interest in natural or synthetic antioxidants in chickens has surged, as avian coccidiosis causes severe inflammation followed by lipid peroxidation of the intestinal mucosa [4]. Indeed, Colnago et al. [31] found that dietary selenium or vitamin E reduced an *Eimeria*-mediated increase in mortality and growth depression in broiler chickens. The latter findings led us expect the anticoccidial activity of antioxidant sulfur (MSM and SS) in experimental avian coccidiosis. In contrast to our expectation, dietary MSM or SS did not affect growth performance nor lower gut lesions in coccidiosis vaccine-challenged broiler chickens, although both MSM and SS produced antioxidant activity at 21 days (i.e., before coccidiosis vaccine challenge). In line with our study, the antioxidant activity of MSM and SS in laying hens [20] and MSM in broiler chickens [17,19] has been reported. In addition, it has been reported that antibiotic plant extracts [5], vitamin E [6], and selenium [7] are known to lessen the gut severity of *Eimeria* infection in broiler chickens. Tentatively, it is tempting to conclude that the antioxidant activity per se might not determine the anticoccidial activity of the potential candidates. Nonetheless, it was perplexing that both MSM and SS tended to increase duodenal lesions while lowering jejunal lesions compared with the challenged control group. In addition, dietary MSM tended to increase cecal lesions compared with the challenged control group. Due to the mild lesions produced by the attenuated coccidiosis vaccine, this marginal increase or decrease in the gut lesions of the sulfur-fed chickens over the challenged control group may not be considered an accurate indicator of anticoccidial activity. Commonly, it is well known that the field isolates of *Eimeria* can cause more severe intestinal damage compared to overdose of attenuated coccidiosis vaccine strains [22]. Whether dietary MSM or SS at different inclusion levels would be more effective in clinical coccidiosis with severe gut lesions waits to be addressed in future studies.

It is well reported that coccidiosis impairs nutrient digestion leading to a low productive performance in broiler chickens. For example, Amerah and Ravindran [32] reported that a coccidia challenge with field isolates resulted in reducing the AID of dry matter, nitrogen, starch, fat, and energy of broiler chickens compared with the naïve control chickens. Of interest, the latter group [32] found that the AID of crude ash was not affected by *Eimeria* infection. Dunaway and Adedokun [33] noted that a coccidiosis vaccine challenge lowered the digestibility of dry matter, nitrogen, and metabolizable energy in broiler chickens compared with the naïve control group. Similarly, we found that coccidiosis vaccine challenge lowered the AID of crude protein and crude ash, albeit that it did not affect that of dry matter. This finding coupled with lowered gut lesions suggests the negative effect of coccidiosis on the gut integrity of chickens. In this study, MSM and SS did not affect the *Eimeria*-induced reduction in the AID of crude protein. However, MSM, but not SS, improved the *Eimeria*-induced reduction in the AID of crude ash. The clear explanation for the improved effect of MSM over SS on ileal ash digestibility is not readily available, but it might be related to mineral interaction or dynamics in gut lumen. Summers et al. [34] found that bone ash contents were linearly lowered with increasing dietary sulfur, indicating that calcium would be precipitated as an insoluble salt with a high sulfate concentration in the intestinal lumen. It would thus be likely that there is an interaction between sulfur and macro/micro minerals in the intestinal lumen. However, as both MSM and SS diets had equal sulfur contents, it might be less likely that the effect of sulfur per se on the mineral dynamics was the main explanation for the MSM vs. SS regarding favoring ash digestibility. Whether MSM vs. SS is more effective in mineral utilization/absorption at the gut level needs to be addressed. The latter statement can be addressed in analyzing macro/micro minerals in digestion trials to see which minerals are interacting with dietary MSM.

The SCFAs are known as the major end-products of fermentation by gut microflora on undigested carbohydrates including non-starch polysaccharides, are used as a nutrient source for colon epithelial cells, and have an inhibitory effect on intestinal pathogenic bacteria [35,36]. It is reported that sulfur is known to have antimicrobial activities in chickens as manifested by improved gut morphology and an increased *Lactobacillus* but lowered *Escherichia coli* [37]. Similarly, Jiao et al. [29] noted that increasing dietary MSM from 0.05 to 0.2% in diets linearly increased *Lactobacillus* but lowered *E. coli* in the excreta of broiler chickens. However, neither MSM nor SS affected cecal SCFAs in chickens in this study, although the former increased the relative percentage of BCFA by an average of 19.2% compared with the control group. The contradictory roles of BCFA have been reported, with both increases [38] decreases in the inflammatory responses [39]. It is however not clear whether the increased cecal BCFA concentration noted in this study could explain the slight increase in cecal lesions in the MSM-fed chickens, as the BCFA concentration remained low from 2.1 to 3.2% of total SCFA. Further studies are needed to delineate the interplay between gut microbiota and local/systemic immunity as highlighted elsewhere [40].

## 5. Conclusions

It is concluded that dietary MSM and SS did not affect performance of broiler chickens. Both MSM and SS increased hepatic antioxidant TAC concentrations. The coccidiosis vaccine induced mild gut lesions and tended to result in lower growth performance. However, dietary MSM and SS did not have any effect on vaccine-induced gut lesions and growth depression compared with the challenged control group. Coccidiosis vaccine challenge decreased the apparent ileal digestibility of crude protein and crude ash. Dietary MSM, but not SS, improved crude ash digestibility compared with the challenged control group. Although both MSM and SS exhibit the antioxidant property, their efficacy as anticoccidial agents to control coccidiosis under the *Eimeria* infection model used here is considered minimal.

## Figures and Tables

**Table 1:** Ingredient and nutrient composition of the basal diet.

Ingredients	g/100 g of Diet
Corn, 7.13% CP	56.90
Soybean meal, 44.7% CP	29.00
Corn gluten meal, 63.8% CP	7.00
Animal fat	2.00
NaCl	0.30
Monocalcium phosphate	1.30
_DL_-methionine, 99%	0.35
_L_-lysine, 56%	0.50
_L_-threonine, 99%	0.10
Ground limestone	1.90
Choline chloride, 50%	0.20
Vitamin premix ^1^	0.20
Mineral premix ^2^	0.25
Total	100.0
Calculated nutrient composition, %	
Nitrogen-corrected apparent metabolizable energy, kcal/kg ^3^	3047
Dry matter ^3^	88.0
Crude protein ^3^	23.7
Calcium ^4^	1.03
Total phosphorus ^4^	0.71
Available phosphorus ^4^	0.45
Lysine ^4^	1.33
Methionine ^4^	0.72
Methionine + Cysteine ^4^	1.08
Threonine ^4^	0.93

^1^ Vitamin mixture provided following nutrients per kg of diet: vitamin A, 9000 IU; vitamin D3, 4000 IU; vitamin E, 58 mg; vitamin K_3_, 2.7 mg; vitamin B_1_, 2.3 mg; vitamin B_2_, 5.9 mg; vitamin B_5_, 17 mg; vitamin B_6_, 2.9 mg; vitamin B_12_, 0.015 mg; niacin, 54 mg; folic acid, 1.7 mg; biotin, 0.16 mg. ^2^ Mineral mixture provided following nutrients per kg of diet: Mn, 85.7 mg; Cu, 100 mg; Zn, 64.3 mg; Fe, 57.1 mg; I, 0.57 mg; Co, 0.17 mg; Se, 0.2 mg. ^3^ Analyzed value. ^4^ Calculated values.

**Table 2:** The analyzed contents of methyl sulfonyl methane (MSM) and sulfur in the experimental diets.

Supplemental Treatment	CONT ^1^	MSM	SS
Sodium bicarbonate, g/kg	4.00	4.00	-
MSM, g/kg	-	2.00	-
SS, g/kg	-	-	3.00
Analyzed			
MSM, g/kg	0.284	1.714	0.292
Sulfur, g/kg	3.52	4.20	4.21

^1^ CONT = challenged control; MSM = methyl sulfonyl methane; SS = sodium sulfate.

**Table 3:** Effect of dietary sulfur sources on growth performance in broiler chickens ^1^.

Item ^2^	NEG ^3^	MSM	SS	SEM ^5^	*p*-Value
BW, g/bird					
Day 0	34.71 ^4^	34.84	34.72	0.17	0.951
Day 21	820.5 ^4^	794.3	820.1	12.77	0.466
BWG, g/day/bird					
Day 0 to 21	785.8 ^4^	759.5	785.4	12.77	0.464
FI, g/day/bird					
Day 0 to 21	1058.9 ^4^	1031.6	1066.8	29.22	0.812
FCR, g:g					
Day 0 to 21	1.348 ^4^	1.351	1.363	0.027	0.948

^1^ Values are least-squares means of 8 replicates unless otherwise stated. ^2^ BW = body weight; BWG = body weight gain; FI = feed intake; FCR = feed conversion ratio. ^3^ NEG = unchallenged group; MSM = methyl sulfonyl methane; SS = sodium sulfate. ^4^ Values are least-squares means of 16 replicates. ^5^ SEM, standard error of the means.

**Table 4:** Effect of dietary sulfur sources on growth performance in coccidiosis vaccine-challenged broiler chickens ^1^.

Item ^2^	NEG ^3^	Coccidiosis Vaccine Challenge			SEM ^4^	*p*-Value
		POS	MSM	SS		
BWG, g/day/bird						
Day 21 to 28	571.4	541.4	527.4	540.1	15.741	0.262
FI, g/day/bird						
Day 21 to 28	841.8	818.2	801.9	814.5	18.597	0.504
FCR, g:g						
Day 21 to 28	1.474	1.514	1.521	1.513	0.014	0.087

^1^ All means are average of 8 pens per treatment. ^2^ BWG = body weight gain; FI = feed intake; FCR = feed conversion ratio. ^3^ NEG = unchallenged group; POS = challenged group; MSM = methyl sulfonyl methane; SS = sodium sulfate. ^4^ SEM, standard error of the means.

**Table 5:** Effect of dietary sulfur sources on the concentrations of cecal short-chain fatty acids (mM/g or % of total) in broiler chickens at 21 d of age ^1^.

Item	NEG ^2^	MSM	SS	SEM ^4^	*p*-Value
mM/g					
Acetate	47.08	46.47	47.11	2.99	0.99
Propionate	4.60	4.06	5.21	0.60	0.64
Isobutyrate	0.50	0.61	0.45	0.04	0.10
Butyrate	11.84	12.07	11.71	1.40	0.99
Isovalerate	0.55	0.50	0.42	0.04	0.15
Valerate	0.70	0.66	0.63	0.07	0.80
Lactate	1.32	1.64	2.11	0.37	0.43
BCFA ^3^	1.75	1.77	1.50	0.10	0.29
SCFA ^3^	66.58	66.00	67.64	4.49	0.98
% of total SCFAs					
Acetate	71.86	71.82	69.58	1.07	0.46
Propionate	6.12	6.00	6.31	0.51	0.95
Isobutyrate	0.81	1.10	0.67	0.10	0.10
Butyrate	17.26	16.31	18.68	1.21	0.61
Isovalerate	0.85	1.01	0.57	0.10	0.10
Valerate	1.05	1.11	0.89	0.06	0.21
Lactate	2.06	2.64	3.30	0.51	0.40
BCFA ^3^	2.71	3.23	2.13	0.22	0.06

^1^ All means are average of 8 pens per treatment. ^2^ NEG = unchallenged group; MSM = methyl sulfonyl methane; SS = sodium sulfate. ^3^ SCFA, short-chain fatty acid (acetate + propionate + butyrate + isobutyrate + isovalerate + valerate + lactate); BCFA, branched-chain fatty acid (isobutyrate + valerate + isovalerate). ^4^ SEM, standard error of the means.

**Table 6:** Effect of dietary sulfur sources on antioxidant parameters in broiler chickens at 21 d of age ^1^.

Item ^2^	NEG ^3^	MSM	SS	SEM ^4^	*p*-Value
Serum					
TAC (mM Trolox equivalents)	0.74	0.73	0.80	0.03	0.571
MDA (µM)	22.64	24.51	22.16	1.69	0.761
Liver					
TAC (nmol/mg of protein)	76.47 ^b^	95.82 ^a^	89.54 ^a^	2.13	0.001
MDA (nmol/mg of protein)	10.41	12.41	10.93	0.70	0.205
Ileum					
TAC (nmol/mg of protein)	20.00	22.36	20.53	2.78	0.881
MDA (nmol/mg of protein)	1.45	1.76	1.60	0.17	0.556

^1^ All means are average of 8 pens per treatment. ^2^ MDA = malondialdehyde; TAC = total antioxidant capacity. ^3^ NEG = unchallenged group; MSM = methyl sulfonyl methane; SS = sodium sulfate. ^4^ SEM, standard error of the means. ^a,b^ Means within the same row having different letters differ significantly at *p* ≤ 0.05.

**Table 7:** Effect of dietary sulfur sources on gut lesion scores in coccidiosis vaccine-challenged broiler chickens ^1^.

Item	NEG ^3^	Coccidiosis Vaccine Challenge			SEM ^4^	*p*-Value
		POS	MSM	SS		
Duodenum ^2^	0.00 ^b^	0.38 ^ab^	0.46 ^a^	0.54 ^a^	0.106	0.006
Jejunum ^2^	0.00 ^b^	0.54 ^a^	0.46 ^ab^	0.25 ^ab^	0.121	0.017
Ceca ^2^	0.00 ^b^	0.29 ^ab^	0.33 ^a^	0.29 ^ab^	0.089	0.047

^1^ All means are average of 8 pens per treatment. ^2^ Lesion were scored on a scale of 0 to 4; zero representing no gross lesions and 4 representing extensive hemorrhage or lesions (depending on the *Eimeria* species). ^3^ NEG = unchallenged group; POS = challenged group; MSM = methyl sulfonyl methane; SS = sodium sulfate. ^4^ SEM, standard error of the means. ^a,b^ Means within the same row having different letters differ significantly at *p* ≤ 0.05.

**Table 8:** Effect of dietary sulfur sources on the apparent ileal digestibility of nutrients in broiler chickens.

Item ^1^	NEG ^2^	Coccidiosis Vaccine Challenge			SEM ^3^	*p*-Value
		POS	MSM	SS		
DM, %	90.3	87.5	89.5	88.1	0.937	0.184
CP, %	81.8 ^a^	72.5 ^b^	73.6 ^b^	71.7 ^b^	1.514	0.001
Ash, %	47.1 ^a^	36.6 ^c^	42.3 ^b^	38.3 ^bc^	1.205	0.001

^1^ DM = dry matter; CP = crude protein. ^2^ NEG = unchallenged group; POS = challenged group; MSM = methyl sulfonyl methane; SS = sodium sulfate. ^3^ SEM, standard error of the means. ^a,b,c^ Means within the same row having different letters differ significantly at *p* ≤ 0.05.

## Data Availability

Not applicable.
